# Quality of Goji Berry Fruit (*Lycium barbarum* L.) Stored at Different Temperatures

**DOI:** 10.3390/foods11223700

**Published:** 2022-11-18

**Authors:** Danial Fatchurrahman, Maria Luisa Amodio, Giancarlo Colelli

**Affiliations:** Dipartimento di Scienze Agrarie, degli Alimenti e dell’Ambiente, Università di Foggia, Via Napoli 25, 71122 Foggia, Italy

**Keywords:** goji berries, shelf-life, postharvest quality, sensorial attributes, freshness, chilling injury

## Abstract

Goji berries are widely known for their outstanding nutritional and medicinal properties; they are usually found in the market as dried fruit or as juice because the fruit has a short shelf-life, and little information is available about its postharvest behavior at low temperatures. This study aimed to determine the storage performance of goji berry fruit by evaluating physicochemical, and sensorial attributes during storage at three different temperatures (0, 5, and 7 °C) for 12 days in a range that has not been extensively studied before. In addition, fruit respiration and ethylene production rates were also measured at the three temperatures. Fruit stored at 0 °C showed the lowest respiration rate and ethylene production (5.8 mg CO_2_ kg^−1^h^−1^ and 0.7 µg C_2_H_4_ kg^−1^h^−1^, respectively); however, at this temperature, the incidence and severity of pitting and electrolytic leakage were the highest. In contrast, 5 °C was found to be the best storage temperature for goji berry fruit; the fruit appeared fresh and healthy, had the highest scores during sensory analysis with an acceptable general impression, and had the lowest amount of damage attributable to chilling injury, with 17.1% fruit presenting with shriveling, 12.5% pitting, 6.7% mold, and 35% electrolytic leakage on day 9 of storage. Storage of goji berries at 7 °C resulted in the lowest marketability and the highest incidence of decay. Significant differences were also found in the phytochemical attributes, vitamin C content, soluble solid content (SSC), titratable acidity (TA), SSC/TA ratio, total polyphenol content, 2,2-diphenylpicrylhydrazy (DPPH), and anthocyanin content. This study revealed that a storage temperature of 5 °C for 9 days is recommended to maintain the quality of fresh goji berry. Thus, broadening the existing knowledge of the postharvest behavior of fresh goji berries; our results can help improve the commercial life of goji berries and ensure high-quality attributes throughout distribution.

## 1. Introduction

Wolfberries or goji berry fruit (*Lycium barbarum* L.), belonging to the family Solanaceae, are widely recognized for their exceptional health benefits [1]. This shrub is native to Asia, primarily the central north region of Ningxia Hui (China), and was introduced to Europe in the 18th century due to its excellent nutritive and medicinal value [2]. Goji berries are particularly rich in nutraceutical compounds (carotenoids, flavonoids, phenolics, vitamins, and minerals) that exhibit antiaging, antitumor, and antioxidant activities in the human body. Generally, goji berries are cooked and processed as teas, soups, or served with meat and vegetables. They are also utilized for juice, tincture, and wine production [3,4,5]. The fruit is also consumed in its dried form or processed in powdered form for medicinal purposes [6]. Given their high perishability, high water content, and susceptibility to damage and rot [7], fresh goji berries are generally only available in areas where they are cultivated. This is also due to the lack of information on the goji berry’s postharvest behavior and storage recommendations [8]. Dipping in lecithin solution [9] and edible coating applications based on lotus leaf extract [7] have been studied to prolong the shelf-life of goji. A study reported that a mild heat treatment (40 °C for 30 min) combined with a chitosan coating could delay decay occurrence in goji berries for up to 28 days at 2 °C [10]. A test on CO_2_-enriched atmospheres concluded that 15–20% of carbon dioxide could help maintain the quality of stored goji berries for up to 14 days [11]. The use of modified atmosphere packaging (MAP), studied by [12], reaching an equilibrium of approximately 10% CO_2_ can help preserve berry weight losses and organoleptic quality while avoiding mold occurrence up to day 13 of storage at 7 °C. The postharvest ripening of goji berries has been controlled using salicylic acid treatment, as this acid is used for inhibiting goji berry postharvest decay [13]. Regardless of postharvest technology, temperature and relative humidity are the first storage conditions to be accurately controlled to preserve quality and allow maximum shelf-life [14,15]. Studies on storage temperature of goji berries have only considered low ranges (mostly at around 0 and 2 °C), and high ranges (around 10 or 20 °C). The effect of storage temperatures of −2, 0, 10, and 20 °C on postharvest quality of fresh goji berries was reported by [1] who concluded that 0 °C is the optimum temperature to maintain the berry’s phytochemical and sensory qualities. However, the large temperature gap among the tested temperatures is to be considered as there is no information on the temperature range between 0 and 10 °C, especially considering that 5 °C is the temperature frequently used in the cold chain of fresh products for transport and sale. As goji berries belong to the Solanaceae family, they might plausibly be chilling-sensitive, such as tomatoes, bell peppers, and eggplants [16,17,18]; however, the quality of fruit stored at 0 °C may show a slower degradation compared to when stored at 10 or 20 °C [1]. Therefore, the objective of the present study was to compare the effects of different chilling storage temperatures between 0 and 7 °C to identify the best condition for maintaining goji berry quality and shelf-life, with particular attention to the possible occurrence of chilling injury symptoms. 

## 2. Materials and Methods

### 2.1. Sample Preparation

Briefly, 3.5 kg of goji berry fruit from ‘Sweet Berry’ cultivar grown in an open field in Castellaneta, a province of Taranto, Italy was conventionally handpicked with a peduncle at the ripeness level five as defined by a previous report [19] to be the recommended stage of harvest since the fruit are completely red, with close to the highest soluble solids content, and full size, thus benefitting from the highest yield with the least loss caused by the high perishability of the fruit even at harvest. At this stage fruit were harvested with maturity indicators in terms of color (hue°), weight, TA, SSC, and firmness were approximately 34.2 ± 0.85°, 0.83 ± 0.09 g, 0.69 ± 0.03%, 23.9 ± 0.2%, and 0.39 ± 0.04 N, respectively. Damaged fruits were removed, leaving 3.3 kg of healthy fruit with homologous dimensions. Approximately 120 g of fruit was used for the initial evaluation; then, the fruits were split into 27 groups (approximately 120 g each) with three replicates for storage at 0, 5, and 7 °C under 95% relative humidity (RH). Quality attributes of goji berry fruit were determined on the day of harvest and after 5, 9, and 12 days of storage.

### 2.2. Physical Quality Attributes

The color of the goji berry fruit was measured by extracting images acquired with a spectral scanner (DV s.r.l., Italy). The CIE L*a*b* color space were used for the color parameters. $Hue{}^{\circ}=arctg^{\frac{b^{*}}{a^{*}}}$ and chromaticity = $\sqrt{a^{*}{}^{2}+b^{*}{}^{2}}$ were calculated from the $a^{*}$ and $b^{*}$ values [20]. Firmness was assessed on 20 fruits for each replicate by applying a compression test to rupture the fruit between two parallel plates using a texture analyzer (TA-XT2^®^, Stable Microsystems, Godalming, UK) and a speed of crosshead at 75 mm min^−1^. The rupture load of the deformation curve was recorded in Newton (N). 

### 2.3. Sensorial Analysis

The sensory evaluation of goji fruit was performed by four trained panel members using a method introduced by [21] for apples, which was then applied to goji berries by [1]. The sensorial properties included firmness, texture, juiciness, sugar–acid ratio, aroma, tastefulness, and general impression. The external properties were shape, size, and color. For each attribute, goji berries were evaluated using a 1–5 anchored scale with 5 = excellent (fresh-like), 4 = very good, 3 = good (limit of marketability), 2 = acceptable (limit of edibility), and 1 = unsatisfying (not edible). The assessment was performed on harvest day using around 200 fruit assessed by each panelist, and 300 fruit each were used from each temperature storage on the evaluation day (after 5, 7, 9, and 12 days of storage). In addition, the incidence of mold and visual damage expressed as a percentage of the total number of fruits for each replicate was recorded.

### 2.4. Electrolytic Leakage

Relative electrolyte leakage (REL) was evaluated based on a previously published method with some modifications [22]. Instead of water, a mannitol isotonic solution was used to avoid osmotic shock [23]; 0.4 mol L^−1^ mannitol was determined to be the optimal concentration after following the procedure suggested by [24]. Approximately 10 fruit slices were taken from each replicate (approximately 3 g in weight) and placed in a centrifuge tube with 25 mL of 0.4 mol L^−1^ mannitol (Sigma–Aldrich, Steinheim, Germany). The electrical conductivity of the bathing mannitol solution was measured with a conductivity meter (CM35, Crison, Carpi, Italy) after 1 min (C1) and 60 min (C60) of incubation with orbital shaking (DAS12500, Intercontinental Equipment, Rome, Italy) at a speed of 60 cycles min^−1^. The samples were then frozen at −20 ℃ for 24 h, and the conductivity (CT) was measured after defrosting for 3 h at 25 ℃. The REL was calculated using the following equation:

$$REL\;\left(\%\right)=\left[\frac{\left(C60-C1\right)}{CT}\right]\times 100$$

### 2.5. Physiological and Metabolic Attributes

The respiration rate (mg CO_2_/kg/h) of goji berries was measured under static conditions as previously described by [25] with some modifications. Briefly, three replicates of 90 g fruit each were put in 150 mL sealed glass containers with a plastic septum for sampling. The containers were initially left open in a temperature- and humidity-controlled room to acclimate the samples (0, 5, and 7 °C). After closing, around 1 mL of gas samples were collected from each container, after the necessary time had passed to accumulate CO_2_ in the headspace reaching a concentration of 0.1–0.2%, and injected into a gas chromatograph (Shimadzu, model 17 A, Kyoto, Japan) equipped with a thermal conductivity detector (200 °C). Separation of CO_2_ was performed using a Carboxen 1006 plot (30 m × 0.53 mm, Supelco, Bellefonte, PA, USA) with a flow of the column at 7 mL min^−1^ and an oven temperature of 180 °C. The calculation of the respiration rate was based on the differences in CO_2_ concentration, the weight of the sample, the container’s free volume, and the elapsed time [26].

Ethylene production (µL C_2_H_4_/kg/h) was measured using the closed system introduced by [25]. The accumulation of C_2_H_4_ in the headspace of the sealed containers was measured using gas samples (2.5 mL) that were injected into a gas chromatograph (model 7890 A, Agilent, Santa Clara, CA, USA) installed with a flame ionization detector (FID, Agilent, Santa Clara, CA, USA). The temperature of the detector was set at 300 °C with the hydrogen and air flow at 45 mL min^−1^ and 400 mL min^−1^, respectively. A metal-packed column 13073-U (Supelco, Bellefonte, PA, USA) was used for the separation of ethylene. Helium as the carrier gas (pressure, 15 psi) was used. The temperature of the oven was set to 120 °C. The concentration of ethylene was then referred to the weight of sample, to the container’s free volume, and to the elapsed time.

### 2.6. Maturity Index

A method introduced by [27] was used for measuring SSC, TA, and pH. Ten goji berries per replicate were homogenized in Ultra Turrax (T18 basic, IKA, Staufen, Germany) and then filtered with two layers of cheesecloth (SWAB 4040, JC NONSTE, Shanghai, China). The obtained juice was used for direct SSC (%) reading using a digital refractometer (PR32-Palette, Atago N1, Tokyo, Japan). TA and pH were measured in 1 g of juice samples using an automatic titrator (T50 M Terminal, Mettler Toledo, Greifensee, Switzerland). The samples were titrated against a 0.1 mol L^−1^ NaOH solution up to a final pH of 8.1; the results are reported as a percentage of citric acid per 100 g sample.

### 2.7. Chemical Composition

#### 2.7.1. Determination of Ascorbic Acid, Dehydroascorbic Acid, and Vitamin C Content

Dehydroascorbic acid (DHAA), ascorbic acid (AA), and total vitamin C contents were assessed by homogenizing 5 g of fruit tissue in Ultra Turrax for 1 min with 5 mL of methanol/water (5:95 *v*/*v*), citric acid (21 g L^−1^), ethylenediaminetetraacetic acid solution (EDTA) (0.5 g L^−1^), and sodium fluoride (NaF) (0.168 g L^−1^). The homogenate was filtered through cheesecloth, and the pH was adjusted to 2.2–2.4 with the addition of 6 mol L^−1^ hydrochloric acid HCl. Centrifugation at 12,000 rpm for 5 min of homogenate was applied. The supernatant was recovered and filtered through a C18 Sep-Pak cartridge (Waters, Milford, MA, USA) and a 0.2 μm cellulose acetate filter. AA and DHAA were evaluated as described by Zapata and Dufour (1992) with little modifications [28]. HPLC analysis was conducted after the derivatization of DHAA into fluorophore 3-(1,2-dihydroxy ethyl) furol [3,4-b] quinoxaline-1-one (DFQ) with 1,2-phenylenediamine dihydrochloride (OPDA). All the of 20 μL samples were analyzed using an HPLC system (Agilent Technologies 1200 Series; Agilent, Waldbronn, Germany) installed with a binary pump and a DAD detector. DFQ and AA separation were acquired on a Zorbax Eclipse XDB- C18 column (150 mm × 4.6 mm; 5 μm particle size; Agilent Technologies, Santa Clara, CA, USA). Methanol (MeOH)/water (H_2_O) (5:95 *v*/*v*) was used as mobile phase, containing 50 mmol L^−1^ potassium dihydrogen phosphate and 5 mmol L^−1^ cetrimide at pH 4.5. The rate of the flow was set at 1 mL min^−1^. The detector was set on wavelengths 348 nm for DHAA and 251 nm for AA. The contents of AA and DHAA are expressed as grams of AA or DHAA/kg f.w. 

#### 2.7.2. Determination of Anthocyanin Content

The anthocyanin content was determined using the method introduced by [29]. Two disks (top cut) were taken from fresh goji berries (approximately 1 mm thick). The area of the disks was calculated using the area of the ellipse formula A=a∗b∗π. Goji fruit disks were then mixed with 3 mL of acidified methanolic solution (10 mL HCl/L) until submerged and treated for 3 h at 25 °C under dark conditions. The anthocyanin level was measured according to the formula introduced by [30]:

$$Anthocyanin=Absorption_{532\;\mathrm{nm}}-0.25\;\left(Absorption_{653\;\mathrm{nm}}\right)$$

Afterward, the molar concentrations of anthocyanins/cm^2^ were obtained by dividing the optical density values by the molecular extinction coefficient of cyanidin (2.45 × 10^4^) L/mol*cm and then again dividing by the area of the leaf disks [31]. The results are expressed as milligrams of cyanidin per cm^2^.

#### 2.7.3. Total Polyphenol Content and Antioxidant Activity

The total polyphenol content was evaluated using 5 g of goji berries homogenized in Ultra Turrax for 1 min in a 30 mL medium containing a 20% water: 80% methanol solution and 2 mmol L^−1^ sodium fluoride. The centrifugation at 9000 rpm for 10 min at 4 °C of homogenate was applied. The method followed a protocol previously used by [32] with slight modifications. Briefly, 100 μL of the extract was mixed with 1.58 mL water, 100 μL of Folin–Ciocalteu reagent, and 300 μL of sodium carbonate solution (200 g L^−1^). The absorbance was read at 725 nm against a blank using a spectrophotometer (UV-1700, Shimadzu Jiangsu, Suzhou, China) after allowing the solution to stand for 2 h. The total polyphenol content was calculated based on the calibration curve of gallic acid and expressed as milligrams of gallic acid per 100 g of fresh weight (mg GA 100 g^−1^ f.w.). The antioxidant assay was performed according to the procedure described by [33] with minor modifications. Fifty microliters of the same extract, opportunely diluted, were pipetted into 0.950 mL of DPPH solution to initiate the reaction. The absorbance was read at 515 nm after 24 h of incubation. Trolox was used as a standard, and the antioxidant activity is presented as grams of Trolox equivalents per kg of fresh weight (g TE kg^−1^ f.w.).

### 2.8. Statistical Analysis

The effects of storage duration and storage temperature were analyzed using a two-way ANOVA, and the significance of differences among means was determined using a Tukey’s test at *p* < 0.05. All calculations were conducted using the statistical software IBM-SPSS 2019 (1 New Orchard Road, Armonk, NY, USA). 

## 3. Results and Discussion 

### 3.1. Physical and Sensory Aspects

The effects of temperature, storage duration, and their interactions of goji berry quality attributes are shown in Table 1.

**Figure 1:**
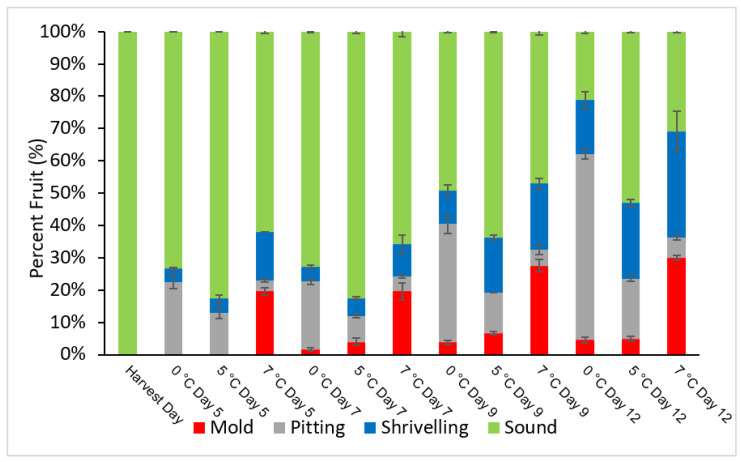
Distribution of fruit alteration types during the storage of goji berries.

The goji berry fruit underwent several types of changes during storage, depending on the temperature, including shriveling, pitting with the appearance of black spots, when severe, and the development of decay. Both storage duration and storage temperature significantly affected shriveling, pitting, and mold occurrence (Table 1). It is reported that mold occurrences in fresh goji berries were most likely caused by Alternaria sp. and many other pathogens, including *Penicillium* sp., *Alternaria* sp., *Aspergillus niger*, *Trichoderma*, and *Aspergillus* [34]. As depicted in Figure 1, all types of alterations were already visible after 7 days of storage, and the most severe mold infection was seen at 7 °C, reaching more than 19.6% versus approximately 2 and 4% at lower temperatures of 0 and 5 °C, respectively. As for shriveling, a higher incidence was observed in berries stored at 5 and 7 °C, reaching approximately 5.7% and 10%, respectively. Although in general fruit stored at 0 and 5 °C showed better physical quality in terms of damage, the fruit possessed a higher amount of pitting at 21.2% and 8.2, respectively, compared to temperature storage at 7 °C which was only around 4.6%. The incidence of damaged fruit gradually increased over time, being a maximum of 30% mold on day 12 of storage at 7 °C, against approximately 5% at 0 and 5 °C.

The information from Figure 1 and the results on sensory attributes reported in Table 2, revealed that goji berries could be stored maintaining a good quality for about 9 days at 5 °C, although 6.7% of the berries had a very early decay appearance indicated by an early spot of mold on the surface of the fruit’s skin. Furthermore, since the highest incidence of pitting was found in goji berries stored at 0 °C where only 49% of the fruit remained sound after 9 days of storage compared to fruit stored at 5 °C with 64% of sound berries, whereas fruit stored at 7 °C, although showing the lowest incidence of pitting, only showed 47.1% of sound fruit. These results indicate that the storage temperature of 5 °C should be preferred over 0 °C. One further consideration is that while berries stored at 0 and 7 °C were characterized by high amounts of alterations which are considered to be unacceptable for marketing (i.e., pitting and mold) in the case of fruit stored at 5 °C the most represented type of alteration was shriveling damage, which could probably be attributed to water loss during storage, and which (unless it is very severe) might not affect the fruit marketability. This is supported by results for sensory evaluation after 9 days of storage (Table 2). As seen in Table 2, the fruit stored at 0 and 5 °C had a reasonable marketable acceptance with a score higher than three (good) for general impression. Among the three storage conditions, after 9 days of storage fruit stored at 5 °C had the highest scores for general impression, tastefulness, aroma, and juiciness reaching 3.3, 3.2, 3.2, and 3.3, respectively. In contrast to fruit stored at 7 °C, which had scores between 2 and 2.5. Although the sensorial attributes of fruits at 0 °C were acceptable, fruits stored at 5 °C showed the highest scores (Table 2). In this experiment, we considered 9 days as the probable shelf life of goji fruit since in the sampling at day 12 the fruit showed scores lower than two for practically all quality attributes, which is very much below the limit of marketability and, in many cases, below the limit of edibility (data not shown). Furthermore, our result is not in accordance with a study by [1] which indicates that the best storage temperature for fresh goji berries is 0 °C when compared to −2, 10, and 20 °C, although in their study storage temperatures between 0 and 7 °C were not explored. 

Color is an important parameter to assess the quality of produce; a bright red–orange color in fresh goji berries is provided by carotenoids [35]. A study reported that fresh mature goji berries contain as much as 321.1 µg.g^−1^ β-carotene [9]. Additionally, among β-carotene types, the content of zeaxanthin esters in goji fruit can exceed 77.5% of βCE/g f.w., and especially zeaxanthin palmitate is found to be abundant, reaching 31–56% of mg βCE/g f.w. [36]. The effect of temperature on accelerating ripening, senescence, color changes, and other attributes is very well known [37]. However, in this experiment, hardly any color change was observed on the berries throughout the storage duration for most of the color primary and secondary attributes (i.e., L*, a*, b*, chroma, and ΔE) (data not shown) although some slight differences in hue angle values were observed at day 12 when fruit held at 7 °C showed a lower hue angle than those stored at 0 °C. Moreover, [1] did not report any difference in color during the storage of goji berries for 12 days where storage temperature varied between −2 and 20 °C. 

In a previously published work [19], goji berries were classified into six developmental stages according to their features in terms of size, color, composition, respiration, and ethylene production rates. While full color was reached already at stage four, fruit size at that stage was still less than 60% of its final size, and many other maturity indicators further evolved during development. Fruits in this experiment were harvested at stage five when they had practically completed their growth and almost reached the maximum SSC. Beyond that stage, the fruit had a limited increase in SSC and no further changes in size and color, although, at stage six the berries resulted very soft and slightly overripe with very poor attributes to go through harvest and postharvest handling procedures. The results of this experiment confirm that ripening completion and beginning of senescence did not affect color changes in the berries nor, as it will be shown below, changes in SSC and TA.

**Figure 2:**
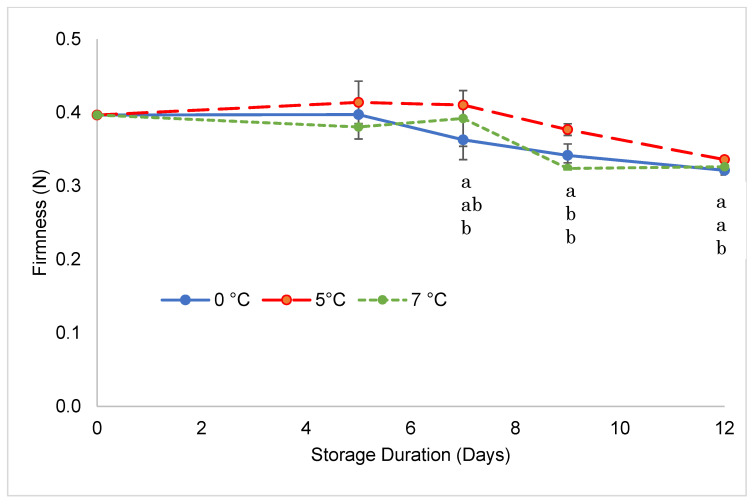
Firmness changes in goji berries during storage at 0, 5, and 7 °C. Values marked with the same letter on the same sampling day are not significantly different, according to a Tukey’s test (*p* < 0.05).

**Figure 3:**
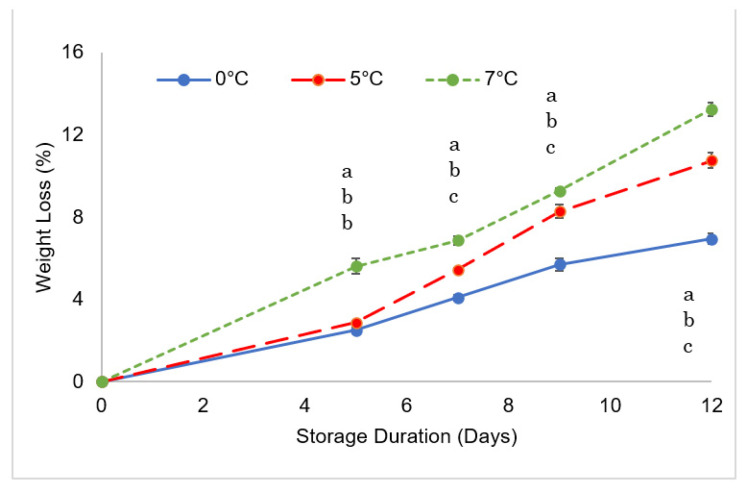
Weight loss changes in goji berries during storage at 0, 5, and 7 °C. Values marked with the same letter on the same sampling day are not significantly different, according to a Tukey’s test (*p* < 0.05).

Fruit firmness showed a limited decrease during storage, although there were some statistically significant differences among treatments (Figure 2). Most softening happened in the second part of storage as values maintained almost constant during the first week of storage. After that fruit stored at 0 °C showed the highest softening rate always presenting significantly lower values than samples at 5 °C. Fruits stored at 7 °C showed a somewhat intermediate behavior but with values that were always closer to samples at 5 °C. While behavior at 5 °C was confirmed by sensorial analysis, as fruit stored at that temperature obtained the highest score for texture, those stored at 7 °C were perceived as the softest by the evaluators, at least after 9 days of storage. Fruit softening is caused by cell wall and middle lamella hydrolysis, where more than 50 genes related to the cell wall structure show variation in expression, involving complex quantitative trait loci [38]. Expression is often regulated by the ripening process and senescence although in many cases fruit texture, as instrumentally determined, is also strongly influenced by tissue turgidity [39]. In a previously published work [19], goji berries showed an important loss of firmness during their development from stage one (green) to stage three (partially red); after that stage fruit firmness remained more or less constant until the overripe stage (six) although the fruit size increased until stage five. This indicates that the loss of firmness during storage observed in this experiment may not be attributed to ripening and senescence. Further, the weight loss of the fruit stored at 0 °C was the lowest (as later described in Figure 3) followed by that of the fruit stored at 5 °C and 7 °C and this also excludes the idea that the faster softening that occurred in fruit stored at 0 °C might be attributed to a more severe loss of turgor. A change in the structure of middle lamellae due to the reduction of electron density has been observed in mature-green tomato fruit that had been chilled at 5 °C for 15 days [40]. The report explained that the regions of reduced electron density could be attributed to water absorption by the cell wall and mediated by pectinmethylesterase alteration of middle-lamellar pectin. Translocation of water from the cytosol is directly associated with an increase in hydration sites in the cell wall and chill-induced membrane dysfunction [41]. The resulting turgor loss, combined with increased water absorption or swelling of cell walls, may be responsible for chilling-associated softening. These results were also in accordance with mechanisms of woolliness development in chilled nectarines [42]. 

As the storage duration increased the fruit’s weight loss, as shown in Figure 3. A significant weight reduction was expected due to the berry’s highly perishable nature; however, the rate of weight loss was greatly influenced by storage temperature, probably because thermal conditions strongly influence the water pressure deficit between the fruit tissue and the surrounding air. Fruit stored at 0 °C showed the lowest weight loss (7% on day 12) which was very significantly lower than the weight loss observed for samples stored at 5 °C (11%) and 7 °C (13%). This result confirmed that at a higher temperature storage of 5 and 7 °C we observed higher incidents of shriveling damage due to water loss (Figure 1). Accordingly, [1] reported that goji berries showed a higher weight loss (18%) when stored at 10 °C than when stored at 0 °C (13%) and concluded that a storage temperature of 0 °C was best for retaining fruit freshness for up to 12 days of shelf-life. 

Fruit respiration rates increase with storage temperature [25]. Accordingly, in this experiment berries stored at 7 °C showed the highest respiration rate (28.2 mg CO_2_/kg/h), while the rates at 5 °C and 0 °C were 13.3 and 5.8 mg CO_2_/kg/h, respectively (Table 3). In addition to the effect of temperature on fruit metabolism, the higher respiration values in fruit stored at 7 °C may result from increased cell damage or microbial growth [43]. The rate of ethylene production of goji berry fruit was at a moderate level when stored at 7 °C (3.8 µg C_2_H_4_/kg*h) and low levels at 0 and 5 °C (0.7 and 0.9 µg C_2_H_4_/kg*h, respectively). Zhou et al. (2020) reported a respiration rate and ethylene production of 80 mg CO_2_/kg/h and 30 µg C_2_H_4_/kg/h, respectively, for goji berries stored at 4 °C [44]; we observed a lower metabolic activity in comparison to these findings. This difference may be attributed to the different cultivars and growing conditions of goji berries used by Zhou et al. (‘Zhongkelvchuan’). Our result is in line with that presented in another study where the respiration rate of goji berries grown in Italy was 27.1 mg CO_2_/kg/h at 7 °C [12].

**Figure 4:**
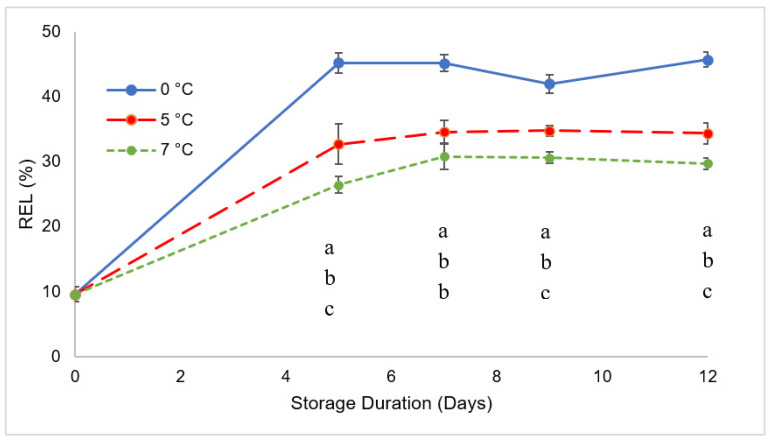
Relative electrolytic leakage of goji berries during storage. Values marked with the same letter on the same sampling day are not significantly different, according to a Tukey’s test (*p* < 0.05).

Figure 4 depicts the electrolytic leakage of the goji berries during storage as being strongly affected by temperature. Electrolytes are contained within membrane-bound compartments in living cells. The proteins and lipids of these membranes are degraded and oxidized under stress (including chilling injury when commodities are sensitive to low temperatures) and during senescence, leading to structural changes that cause loss of integrity and increased membrane permeability [45]. In this case, we may observe a strong effect of storage temperature which influence the changes in REL due to regular senescence. We would have expected that the higher the temperature, the higher the progress in senescence and then the higher the rate of membrane loss of functionality. In this experiment although REL increased for all three treatments after 5 days of storage, the increase was only 2-fold in samples at 7 °C, while samples stored at 5 and 0 °C showed 3- and 4-fold increases, respectively. In addition, while samples stored at 5 and 7 °C showed a further increase in the following 2 days of storage, the value reached in samples stored at 0 °C did not increase with storage time. 

These findings may suggest that while low-temperature damage to membranes was so severe after only 5 days of storage at 0 °C, it may have been caused by biochemical changes in the bilayers which occurred after the primary chilling temperature storage and that may contribute to the development of chilling symptoms reflected by the activated phospholipid catabolism and a consequent accumulation of free fatty acids in the membrane bilayers, contributing to membrane deterioration, and a loss of compartmentation as previously reported in tomatoes [45]. It did not grow any worse as a consequence of tissue senescence, whereas in the other two treatments the damage became more severe after at least 7 days of storage (never reaching the levels showed by berries stored at 0 °C) which indicates it is less likely to be attributed to chilling and more likely to be a result of tissue senescence. Furthermore, it should be taken into consideration that goji berries (as it happens to many other fruits of the Solanaceae family) might be sensitive to temperatures below 10 °C, although the severity of chilling injury symptoms increases with decreasing temperatures. In addition, goji berries are harvested when fully ripe and, at this stage, might better withstand higher ranges of chilling temperatures (e.g., from 5 to 10 °C, such as red tomatoes) as the severity of chilling injury is two times greater in mature green fruits than pink and red fruits at 5 °C [46]. while even a few days of storage at lower ranges might be very critical in terms of quality maintenance.

### 3.2. Chemical and Nutritional Aspects

**Figure 5:**
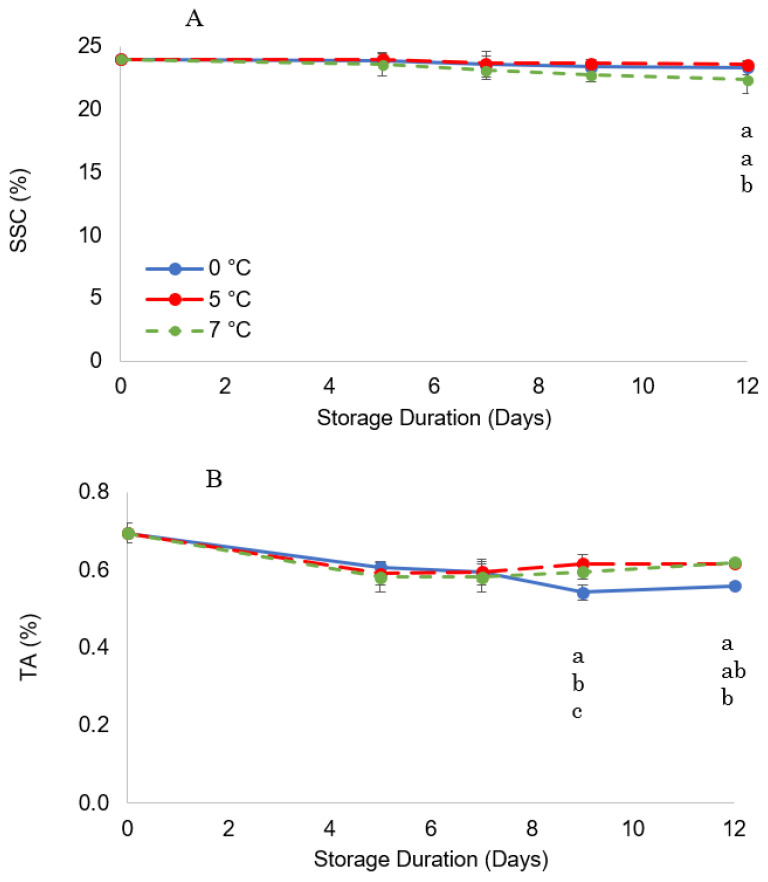
Change in soluble solid content (SSC) (**A**) and titratable acidity (TA) (**B**) during storage. Values marked with the same letter on the same harvest day are not significantly different, according to a Tukey’s test (*p* < 0.05).

Soluble solids slightly decreased during storage (Figure 5A) with the decrease rate at 7 °C significantly higher than that at 0 °C, reaching 22.3% after 12 days, while samples at lower temperatures maintained closer values to the initial values. These results are in line with the findings obtained by [10], who found a decrease in total soluble content from 21 (initial) to 17.32% during storage in the control samples of Chinese wolfberry fruit stored at 2 ± 0.5 °C. The berries used in this experiment had already completed growth, reached full color, and had almost reached maximum values of SSC while on the plant (stage five); a postharvest decrease of SSC in fruit at this stage could be expected due to high respiration and senescence which can be better observed in fruit at 7 °C. On the other hand, harvest at an earlier stage was hardly an option since fruit at stage four have not reached full size yet, thus representing a potentially severe decrease in fruit yield [19]. Nonetheless, the maximum SSC reported for the maturity stage in this study was 23.5%, which remained constant over the 12-day storage period and low storage temperatures delayed senescence, keeping SSC almost unchanged for samples at 5 and 0 °C.

The TA of goji berry fruit changed during storage; namely from 0.7% on harvest day it decreased to about 0.56% at 0 °C and 0.62% at 5 and 7 °C at the end of storage (Figure 5B). Such changes, particularly evident at 0 °C, could be caused by the loss of organic acids and minerals because of chilling stress, as also seen in tomatoes [47]. In addition, the higher percentage of water loss observed in samples at 5 and 7 °C could be the reason for the higher values of TA compared to samples stored at 0 °C, as also observed for SSC in raspberry fruit [48]. These results were reflected in the SSC/TA ratio trend, which at 12 days was highest for fruits stored at 0 °C (41.6) compared with that of fruits stored at 5 and 7 °C (38.2 and 36.2, respectively), as shown in Table 4.

**Figure 6:**
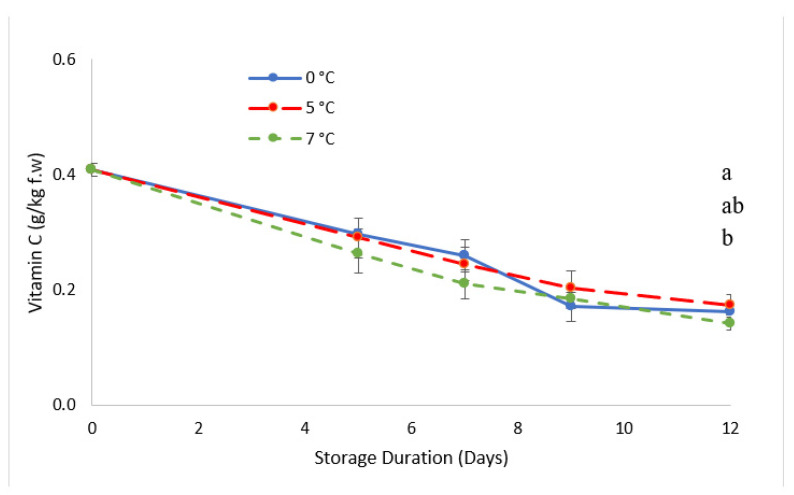
Changes in vitamin C levels during storage. Values marked with the same letter on the same harvest day are not significantly different according to a Tukey’s test (*p* < 0.05).

Goji berries are rich in vitamin C, which is beneficial for human health [49]. Figure 6 depicts the vitamin C level as the sum of the contents in AA and DHAA (which are shown in Table 4). Vitamin C decreased during storage for 12 days under all temperature conditions; in particular, it varied from 0.408 to 0.142 g/kg f.w. for fruit stored at 7 °C, which was significantly lower than that for the sample stored at 5 °C (0.175 g/kg f.w.), while samples stored at 0 °C showed an intermediate content (0.163 g/kg f.w.). Goji berries stored at 7 °C showed the lowest vitamin C levels, most probably because of the high levels of oxidation occurring at higher temperatures, as indicated by the higher value of the DHAA content throughout storage. Ascorbic acid is subject to oxidative and enzymatic degradation to dehydroascorbic acid. Ascorbic oxidase is the endogenous enzyme involved in this process [50]. Higher temperature storage might be responsible for the higher oxidation resulting in the higher level of dehydroascorbic acid, which is explained in the results of a study on goji berries which followed the results on kiwi fruits comparing three levels of storage temperature (0, 5, and 10 °C) for 6 days, concluding that the level of DHAA became higher as the temperature storage increased [51].

The higher vitamin C reduction observed at 9 days of storage at 0 °C compared to that at 5 °C may be explained by an intense oxidase activity influenced by the drop of pH (data not shown) as described previously, where the lower pH affects the degradation of vitamin C [52] which may be due to chilling injury, and which is also reported in tomatoes [53].

Our results are consistent with those presented by [54]; they reported that goji berries contain approximately 40 mg/100 g f.w. of vitamin C on the day of harvest, which is comparable with the content of citrus fruits. Therefore, they should be stored in proper conditions since the content is halved after 7 days of storage.

A significant effect of storage temperature was observed in the total polyphenol content of goji berries. Fruit stored at 0, 5, and 7 °C respectively presented values of 2.55, 2.25, and 2.13 g/kg after 12 days (Table 4). The lower levels of total polyphenol at 5 and 7 °C may be attributed to the higher oxidation rates caused by higher temperatures [55]. The range and abundance of phenolic compounds in fruit may vary depending on geographical location, genetic variation, agricultural practices, year of harvest, growth period, or storage conditions [56]. In [54], the authors reported that the content of polyphenolic compounds in *Lycium* spp. was 9.41 g/kg f.w., whereas the authors in [57] reported this value to be 1.42 g/kg.

The DPPH antioxidant activity was significantly lower in fruits stored at 0 °C (2.74 g Trolox/kg) than in those stored at 5 °C (2.97 g Trolox/kg) and 7 °C (3.216 g Trolox/kg) on day 12 (Table 4). This may be due to the contribution of the secondary metabolites zeaxanthin and β-carotene—abundant in goji berry fruit which is best conserved at temperatures 7–10 °C [2]. This result is consistent with results presented by [1], where goji berries stored at 10 °C for 12 days showed a higher DPPH antioxidant activity level (2.8 g Trolox/kg) compared with levels in fruits stored at 0 °C (2.3 g Trolox/kg). Furthermore, a significant difference in DPPH activity was observed based on the day of storage, as the level of antioxidant activity increased from the initial day.

Finally, in this study, we did not observe any significant differences in terms of total anthocyanins (expressed as cyanidin) content in goji fruits in relation to storage temperatures which is in accordance with a previous report [1]. Although, it is reported that low temperature induced anthocyanin accumulation on tomato leaves [58] and eggplant during storage at 2 °C [18].

## 4. Conclusions

Storage temperature is a key factor for the proper storage and handling of fruits, especially for those which are very perishable after harvest such as goji berries. This study complements existing information on the effect of low-storage temperature on goji fruit quality and storability. Our study indicated that 5 °C should be recommended as the appropriate storage temperature over 0 and 7 °C since it induced the lowest level of physiological disorders including pitting, and shriveling while preserving overall sensorial and nutritional quality attributes including vitamin C levels, soluble solids content, and antioxidant activity for 9 days. Although the level of mold was a little higher at 5 °C compared to 0 °C, diffuse chilling symptoms were observed at 0 °C in the form of pitting. These findings indicated that goji fruit may be stored for about 9 days at 5 °C, but additional technologies such as modified atmosphere packaging may be needed to better control decay and to allow safe distribution and consumption.

## Figures and Tables

**Table 1:** Effect of storage temperature, storage duration, and interaction on quality attributes of goji berries stored for 12 days at 0, 5, and 7 °C. Mean values of 12 samples are reported (three replicates × four storage durations).

Quality Attributes	Storage Temperature	Storage Duration	Storage Temperature × Storage Duration
Weight loss (%)	****	****	****
Hue (°)	**	**	ns
Chroma	*	***	*
Firmness (N)	***	ns	ns
Mold damage (%)	****	****	****
Shriveling damage (%)	****	****	****
Pitting (%)	****	***	***
Relative Electrolyte Leakage (%)	****	*	*
Soluble Solid Content (%)	****	****	****
Soluble Solid Content Total Acidity	*	*	ns
Total Acidity (%)	*	ns	*
Ascorbic Acid (g/kg)	****	****	ns
Dehydroascorbic Acid (g/kg)	****	ns	ns
Vitamin C (g/kg)	*	****	ns
Anthocyanin (mg cyanidin cm^−2^)	****	****	*
Total polyphenol (g gallic acid/kg)	****	****	****
Antioxidant (g Trolox/kg)	****	****	****
Firmness (score)	***	****	ns
Texture (chewing; score)	****	****	ns
Juiciness (score)	****	****	ns
Sugar–acid ratio (score)	****	****	ns
Aroma (score)	****	****	ns
Tastefulness (score)	****	****	ns
General impression (score)	****	****	ns
Shape (score)	****	****	ns
Size (score)	****	****	ns
Color (score)	****	****	*

Note: (****) *p* ≤ 0.0001; (***) *p* ≤ 0.001; (**) *p* ≤ 0.01; (*) *p* ≤ 0.05; ns, not significant.

**Table 2:** Sensory evaluation of goji berries stored at three different temperatures for 9 days. A 1–5 anchored scale was used with 5 = excellent (fresh-like), 4 = very good, 3 = good (limit of marketability), 2 = acceptable (limit of edibility), and 1 = unsatisfying (not edible). In each line, values marked with the same letter are not significantly different according to a Tukey’s test (*p* < 0.05).

Sensory Attributes	0 °C	5 °C	7 °C
Firmness	3.2a	3.3a	2.3b
Texture	3.0a	3.2a	2.3b
Juiciness	3.0b	3.3a	2.2c
Sugar–Acid Ratio	3.2a	3.2a	2.3b
Aroma	2.8b	3.2a	2.3c
Tastefulness	2.7b	3.2a	2.5c
General Impression	3.0b	3.3a	2.3c
Shape	3.2a	3.2a	2.3b
Size	3.2a	3.2a	2.3b
Color	3.0a	3.0a	2.0b

**Table 3:** Respiration rate and ethylene production in goji berries at different temperatures using five replicates of each temperature measurement.

Temperature	Respiration Rate mg CO_2_/kg/h	Standard Error (SE)
7 °C	28.2	3.4
5 °C	13.3	0.8
0 °C	5.8	0.8
**Temperature**	**Ethylene Production** µg C_2_H_4_/kg*h	**Standard Error (SE)**
7 °C	3.8	0.1
5 °C	0.9	0.2
0 °C	0.7	0

**Table 4:** Chemical and nutritional quality of goji berries stored for up to 12 days at 0, 5, and 7 °C. SSC = Soluble solids content; TA = Titratable acidity; AA = Ascorbic acid; DHAA = Dehydroascorbic acid; TP = Total polyphenols; AoxA = Antioxidant activity; AC = Anthocyanin content.

		SSC/TA	AA (g kg^−1^)	DHAA (g kg^−1^)	TP (g gallic Acid kg^−1^)	AoxA (g Trolox kg^−1^)	AC (mg cyanidin-3-glucoside cm^−2^)
Day 0		34.61 ± 1.449	0.254 ± 0.023	0.155 ± 0.015	2.995 ± 0.051	2.157 ± 0.087	1.185 ± 0.03
Day 5	0 °C	39.27 ± 0.45a	0.221 ± 0.01a	0.075 ± 0.006a	2.592 ± 0.014b	1.834 ± 0.039b	1.263 ± 0.389a
	5 °C	40.59 ± 1.11a	0.18 ± 0.01b	0.111 ± 0.043a	2.693 ± 0.071a	1.897 ± 0.025b	0.978 ± 0.412a
	7 °C	40.59 ± 3.67a	0.115 ± 0.01c	0.147 ± 0.029a	2.716 ± 0.009a	2.269 ± 0.08a	1.286 ± 0.441a
Day 7	0 °C	39.73 ± 1.59a	0.193 ± 0.02a	0.067 ± 0.036b	2.352 ± 0.033b	2.106 ± 0.021b	0.853 ± 0.124a
	5 °C	39.76 ± 1.98a	0.14 ± 0.02b	0.105 ± 0.012ab	2.750 ± 0.033a	2.322 ± 0.035a	0.813 ± 0.342a
	7 °C	39.77 ± 2.39a	0.076 ± 0.02c	0.134 ± 0.004a	2.395 ± 0.062b	2.336 ± 0.059a	1.098 ± 0.317a
Day 9	0 °C	43.15 ± 1.68a	0.122 ± 0.032a	0.049 ± 0.008a	2.711 ± 0.063a	2.106 ± 0.07a	0.983 ± 0.203a
	5 °C	38.49 ± 1.76b	0.091 ± 0.023ab	0.112 ± 0.007b	2.374 ± 0.071b	2.322 ± 0.028a	0.857 ± 0.326a
	7 °C	38.33 ± 1.59b	0.052 ± 0.019b	0.133 ± 0.004c	2.258 ± 0.029b	2.336 ± 0.092a	1.159 ± 0.093a
Day 12	0 °C	41.63 ± 0.76a	0.091 ± 0.01a	0.073 ± 0.013b	2.545 ± 0.083a	2.746 ± 0.054b	1.078 ± 0.523a
	5 °C	38.20 ± 0.87b	0.049 ± 0.01b	0.125 ± 0.017a	2.224 ± 0.067b	2.971 ± 0.064ab	1.361 ± 1.028a
	7 °C	36.17 ± 1.46b	0.02 ± 0.01c	0.122 ± 0.007a	2.126 ± 0.054b	3.216 ± 0.082a	0.917 ± 0.168a

In the columns, on the same sampling day, values marked with the same letter are not significantly different according to a Tukey’s test (*p* < 0.05).

## Data Availability

The data presented in this study are available on request from the corresponding author.

## References

[1] Jatoi M.A., Fruk M., Buhin J., Vinceković M., Vuković M., Jemrić T. (2018). Effect of Different Storage Temperatures on Storage Life, Physico-chemical and Sensory Attributes of Goji Berry (*Lycium barbarum* L.) Fruits. Erwerbs-Obstbau.

[2] Kulczyński B., Gramza-Michałowska A. (2016). Goji Berry (*Lycium barbarum*): Composition and Health Effects—A Review. Pol. J. Food Nutr. Sci..

[3] Amagase H., Farnsworth N.R. (2011). A review of botanical characteristics, phytochemistry, clinical relevance in efficacy and safety of *Lycium barbarum* fruit (Goji). Food Res. Int..

[4] Benzie I., Wachtel-Galor S. (2011). Herbal Medicine: Biomolecular and Clinical Aspects.

[5] Potterat O. (2011). Goji (*Lycium barbarum* and *L. chinense*): Phytochemistry, pharmacology and safety in the perspective of traditional uses and recent popularity. Planta Med..

[6] Zhu Y., Zi G.Q. (1998). Chinese Materia Medica Chemistry, Pharmacology and Applications.

[7] Fan X.J., Zhang B., Yan H., Feng J.T., Ma Z.Q., Zhang X. (2019). Effect of lotus leaf extract incorporated composite coating on the postharvest quality of fresh goji (*Lycium barbarum* L.) fruit. Postharvest Biol. Technol..

[8] Ling L., Zhao Y., Tu Y., Yang C., Ma W., Feng S., Lu L., Zhang J. (2021). The inhibitory effect of volatile organic compounds produced by Bacillus subtilis CL2 on pathogenic fungi of wolfberry. J. Basic Microbiol..

[9] Jatoi M.A., Jurić S., Vidrih R., Vinceković M., Vuković M., Jemrić T. (2017). The effects of postharvest application of lecithin to improve storage potential and quality of fresh goji (*Lycium barbarum* L.) berries. Food Chem..

[10] Ban Z., Wei W., Yang X., Feng J., Guan J., Li L. (2015). Combination of heat treatment and chitosan coating to improve postharvest quality of wolfberry (*Lycium barbarum*). Food Sci. Technol..

[11] Kafkaletou M., Christopoulos M.V., Tsantili E. (2017). Short-term treatments with high CO_2_ and low O_2_ concentrations on quality of fresh goji berries (*Lycium barbarum* L.) during cold storage. J. Sci. Food Agric..

[12] Palumbo M., Capotorto I., Cefola M., Burbaci S., Pace B. (2020). Modified atmosphere packaging to improve the shelf-life of Goji berries during cold storage. Adv. Hortic. Sci..

[13] Zhang H., Ma Z., Wang J., Wang P., Lu D., Deng S., Lei H., Gao Y., Tao Y. (2021). Treatment with exogenous salicylic acid maintains quality, increases bioactive compounds, and enhances the antioxidant capacity of fresh goji (*Lycium barbarum* L.) fruit during storage. LWT.

[14] Fatchurrahman D., Kuramoto M., Al Riza D.F., Ogawa Y., Suzuki T., Kondo N. (2020). Fluorescence time series monitoring of different parts of green pepper (*Capsicum annuum* L.) under different storage temperatures. Comput. Electron. Agric..

[15] Mastrandrea L., Amodio M.L., de Chiara M.L.V., Pati S., Colelli G. (2017). Effect of temperature abuse and improper atmosphere packaging on volatile profile and quality of rocket leaves. Food Packag. Shelf Life.

[16] Park M.H., Sangwanangkul P., Choi J.W. (2018). Reduced chilling injury and delayed fruit ripening in tomatoes with modified atmosphere and humidity packaging. Sci. Hortic..

[17] Fatchurrahman D., Kuramoto M., Kondo N., Ogawa Y., Suzuki T. Identification of UV-fluorescence components associated with and detection of surface damage in Green Pepper (*Capsicum annum* L.). Proceedings of the 23rd International Conference in Central Europe on Computer.

[18] Tsouvaltzis P., Babellahi F., Amodio M.L., Colelli G. (2020). Early detection of eggplant fruit stored at chilling temperature using different non-destructive optical techniques and supervised classification algorithms. Postharvest Biol. Technol..

[19] Fatchurrahman DAmodio M.L., de Chiara M.L.V., Mastrandrea L., Colelli G. (2022). Characterization and postharvest behavior of goji berry (*Lycium barbarum* L.) during ripening. Postharvest Biol. Technol..

[20] Fatchurrahman D., Amodio M.L., de Chiara M.L.V., Chaudhry M.M.A., Colelli G. (2020). Early discrimination of mature-and immature-green tomatoes (*Solanum lycopersicum* L.) using fluorescence imaging method. Postharvest Biol. Technol..

[21] Miller S., Hampson C., Mc New R., Berkett L., Brown S., Clements J., Crassweller R., Garcia E., Greene D., Greene G. (2005). Performance of apple cultivars in the 1995 NE-183 regional project planting: III. fruit sensory characteristics. J. Am. Pomol. Soc..

[22] Navarro-Rico J., Martínez-Hernández G.B., Artés F., Artés-Hernández F., Gómez P.A. (2015). Effect of edible coatings and electrolyzed water sanitation on fresh-cut “bimi” broccoli quality. Acta Hortic..

[23] Saltveit M.E. (2002). The rate of ion leakage from chilling-sensitive tissue does not immediately increase upon exposure to chilling temperatures. Postharvest Biol. Technol..

[24] Peng J., Tang J., Barrett D.M., Sablani S.S., Powers J.R. (2014). Kinetics of carrot texture degradation under pasteurization conditions. J. Food Eng..

[25] Kader A.A. (2002). Methods of gas mixing, sampling and analysis. Postharvest Technology of Horticultural Crops.

[26] Caleb O.J., Mahajan P.V., Opara U.L., Witthuhn C.R. (2012). Modelling the respiration rates of pomegranate fruit and arils. Postharvest Biol. Technol..

[27] Fatchurrahman D., Nosrati M., Amodio M.L., Chaudhry M.M.A., de Chiara M.L.V., Mastrandrea L., Colelli G. (2021). Comparison performance of visible-nir and near-infrared hyperspectral imaging for prediction of nutritional quality of goji berry (*Lycium barbarum* L.). Foods.

[28] Zapata S., Dufour J. (1992). Ascorbic, dehydroascorbic and isoascorbic acid simultaneous determinations by reverse phase ion interaction HPLC. J. Food Sci..

[29] Proctor J.T.A. (1974). Color Stimulation in Attached Apples With Supplementary Light. Can. J. Plant Sci..

[30] Wells R. (1995). Photosynthetic responses to cutout. Proceedings of the Beltwide Cotton Conference.

[31] Siegelman H.W., Hendricks S.B. (1957). Photocontrol of anthocyanin formation in turnip and red cabbage seedlings. Plant Physiol..

[32] Derossi A., Mastrandrea L., Amodio M.L., De Chiara M.L.V., Colelli G. (2016). Application of multivariate accelerated test for the shelf life estimation of fresh-cut lettuce. J. Food Eng..

[33] Capotorto I., Amodio M.L., Diaz M.T.B., de Chiara M.L.V., Colelli G. (2018). Effect of anti-browning solutions on quality of fresh-cut fennel during storage. Postharvest Biol. Technol..

[34] Lan P., Gao F.R., Chen C.K., Wang W.S., Han J., Ji H.P., Yu J. (2014). Separation and identification of pathogenic fungi from the postharvest *Lycium barbarum*. China Fruit Veg..

[35] Zhang Q., Chen W., Zhao J., Xi W. (2016). Functional constituents and antioxidant activities of eight Chinese native goji genotypes. Food Chem..

[36] Peng Y., Ma C., Li Y., Leung K.S.-Y., Jiang Z.-H., Zhao Z. (2005). Quantification of Zeaxanthin Dipalmitate and Total Carotenoids in *Lycium* Fruits (Fructus *Lycii*). Plant Foods Hum. Nutr..

[37] Kasmire R.F., Kader A.A. (1978). Handling Tomatoes at Wholesale and Retail: A Guide For Better Quality and Greater Profits. Outlook.

[38] Seymour G.B., Chapman N.H., Chew B.L., Rose J.K.C. (2013). Regulation of ripening and opportunities for control in tomato and other fruits. Plant Biotechnol. J..

[39] Toivonen P.M.A., Brummell D.A. (2008). Biochemical bases of appearance and texture changes in fresh-cut fruit and vegetables. Postharvest Biol. Technol..

[40] Marangoni A.G., Jackman R.L., Stanley D.W. (1995). Chilling-Associated Softening of Tomato Fruit is Related to Increased Pectinmethylesterase Activity. J. Food Sci..

[41] Jackman R.L., Gibson H.J., Stanley D.W. (1992). Effects of chilling on tomato fruit texture. Physiol. Plant..

[42] Dawson D.M., Melton L.D., Watkins C.B. (1992). Cell Wall Changes in Nectarines (*Prunus persica*). Plant Physiol..

[43] Rodoni L.M., Feuring V., Zaro M.J., Sozzi G.O., Vicente A.R., Arena M.E. (2014). Ethylene responses and quality of antioxidant-rich stored barberry fruit (*Berberis microphylla*). Sci. Hortic..

[44] Zhou Y., Lai Y., Chen Z., Qu H., Ma S., Wang Y., Jiang Y. (2020). Evolution of physiological characteristics and nutritional quality in fresh goji berry (*Lycium barbarum*) stored under different temperatures. J. Food Process. Preserv..

[45] Campos P.S., Quartin V nia Ramalho J chicho Nunes M.A. (2003). Electrolyte leakage and lipid degradation account for cold sensitivity in leaves of *Coffea* sp. plants. J. Plant Physiol..

[46] Kalantari S., Hatami M., Delshad M. (2015). Diverse postharvest responses of tomato fruits at different maturity stages to hot water treatment. Int. J. Hortic. Sci. Technol..

[47] Ibrahim R., Rhani S.A., Buhri A. (2013). Reduction of chilling injury in tomato (*Solanum lycopersicum*) using different postharvest (pre-storage) treatments. Acta Hortic..

[48] Robbins J.-A., Sjulin T.M., Patterson M. (1989). Postharvest storage characteristics and respiration rates in five cultivars of red raspberry. HortScience.

[49] González-Molina E., Domínguez-Perles R., Moreno D.A., García-Viguera C. (2010). Natural bioactive compounds of Citrus limon for food and health. J. Pharm. Biomed. Anal..

[50] Saari N.B., Fujita S., Miyazoe R., Okugawa M. (1995). Distribution of ascorbate oxidase activities in the fruits of family cucurbitaceae and some of their properties. J. Food Biochem..

[51] Lee S.K., Kader A.A. (2000). Preharvest and postharvest factors influencing vitamin C content of horticultural crops. Postharvest Biol. Technol..

[52] Gil-Izquierdo A., Gil M.I., Conesa M.A., Ferreres F. (2001). The effect of storage temperatures on vitamin C and phenolics content of artichoke (*Cynara scolymus* L.) heads. Innov. Food Sci. Emerg. Technol..

[53] Stevens R., Page D., Gouble B., Garchery C., Zamir D., Causse M. (2008). Tomato fruit ascorbic acid content is linked with monodehydroascorbate reductase activity and tolerance to chilling stress. Plant Cell Environ..

[54] Donno D., Beccaro G.L., Mellano M.G., Cerutti A.K., Bounous G. (2015). Goji berry fruit (*Lycium* spp.): Antioxidant compound fingerprint and bioactivity evaluation. J. Funct. Foods.

[55] Réblová Z. (2012). Effect of temperature on the antioxidant activity of phenolic acids. Czech J. Food Sci..

[56] Dong J.Z., Wang S.H., Zhu L., Wang Y. (2012). Analysis on the main active components of *Lycium barbarum* fruits and related environmental factors. J. Med. Plants Res..

[57] Wang C.C., Chang S.C., Inbaraj B.S., Chen B.H. (2010). Isolation of carotenoids, flavonoids and polysaccharides from *Lycium barbarum* L. and evaluation of antioxidant activity. Food Chem..

[58] Liu Y., Tikunov Y., Schouten R.E., Marcelis L.F.M., Visser R.G.F., Bovy A. (2018). Anthocyanin biosynthesis and degradation mechanisms in Solanaceous vegetables: A review. Front. Chem..

